# Intraperitoneal development of the filarial nematode *Brugia malayi* in the Mongolian jird (*Meriones unguiculatus*)

**DOI:** 10.1007/s00436-014-3829-5

**Published:** 2014-03-25

**Authors:** Yasen Mutafchiev, Odile Bain, Zachary Williams, John W. McCall, Michelle L. Michalski

**Affiliations:** 1 Department of Animal Diversity and Resources, Institute of Biodiversity and Ecosystem Research, Bulgarian Academy of Sciences, Sofia, Bulgaria; 2 Parasitologie Comparée et Modèles Expérimentaux, Muséum National d’Histoire Naturelle, Paris, France; 3 Department of Biology and Microbiology, University of Wisconsin Oshkosh, Oshkosh, WI USA; 4 Department of Infectious Diseases, College of Veterinary Medicine, University of Georgia, Athens, GA USA

**Keywords:** *Brugia malayi*, *Brugia pahangi*, *Meriones unguiculatus*, *Wuchereria bancrofti*

## Abstract

In the present study, we describe intraperitoneal development of the FR3 strain of *Brugia malayi* in Mongolian jirds (*Meriones unguiculatus*). The third molt for male worms occurred between 4 and 7 days postinfection (dpi) and between 4 and 8 dpi for females. The fourth and final molt occurred between days 21 and 29 for males and 25 and 34 for females, considerably earlier than the times reported for subcutaneous infection models using cats and jirds. The timing of the third molt coincided largely with reports for subcutaneous *Brugia pahangi* infections of cats and jirds, but the final molt occurred considerably later and lasted longer than those reported for subcutaneous *B. pahangi* models. Spermatogenesis occurred by at least 50 dpi in adult males, and insemination of females likely occurred between 50 and 60 dpi. Microfilariae were observed in the uteri and ovejectors of adult females at 65 dpi.

## Introduction

*Wuchereria bancrofti* (Cobbold, 1877), *Brugia malayi* (Brug, 1927), and *Brugia timori* (Partono, Purnomo, Dennis, Atmosoedjono, Oemijati and Cross, 1977) are filarial nematodes that cause the mosquito-borne tropical disease lymphatic filariasis (LF). The primary organism currently used for laboratory study of LF is *B. malayi* because the entire life cycle can be propagated experimentally. Development of *B. malayi* in humans is most frequently modeled in the domestic shorthair cat (*Felis catus*) or in the Mongolian jird (*Meriones unguiculatus*), commonly referred to as the Mongolian gerbil, with the jird model being most popular because they are easier to handle and more economical to maintain than cats. Patent infections are produced by injecting infective third-stage larvae (L3) of *B. malayi* subcutaneously (SQ) into jirds. The larvae mature in the lymphatics and the adults produce microfilariae (MF) that circulate in the blood in a manner similar to other susceptible hosts and humans (Ash and Riley 1970a, Ash and Riley 1970b). Alternatively, jirds can be infected by injecting L3s directly into the peritoneal cavity, thus confining larval development and MF production to the peritoneal cavity for facile worm recovery (McCall et al. 1973).

The course of SQ *Brugia* infections in cats and jirds is well described (Ash and Riley 1970a; Ash and Riley 1970b; Ash 1973; Edeson and Buckley 1959; el-Bihari and Ewert 1973; Ewert and el-Bihari 1971). The use of jirds for intraperitoneal (IP) infections with B. pahangi has been described (McCall et al. 1973), and IP-derived *B. pahangi* and *B. malayi* have been widely used for decades, however the developmental timing for neither species has been reported for the IP model. This is surprising because the majority of *B. malayi* and its sister species *B. pahangi* that are used for molecular, immunologic, and -omics studies are generated using the IP jird model. It is clear that IP development of *B. malayi* must be characterized with respect to the development of key anatomic features and biological processes such as molting and reproduction. Here, we report a morphological temporal study of IP *B. malayi* development in the jird host and demonstrate that the third molt occurs slightly sooner than reported for SQ *B. malay*i infection and that the asynchronous fourth molt is earlier and longer in duration than that reported in SQ infected jirds. We also describe the morphological features that were most easily recognized to determine developmental stage and sex for fixed and live *B. malayi*.

## Materials and methods

### Parasite collection and examination

Adult male jirds were IP infected with 150 freshly isolated FR3 strain *B. malayi* L3s at UW Oshkosh using standard methods (Michalski et al. 2011; McCall et al. 1973). All animal manipulations were performed with approved UWO IACUC protocols. Parasites were removed at various time points (3–10, 21–32, 34–38, 51, 60, 65, and 76 days postinfection [dpi]) from euthanized jirds by IP lavage using RPMI culture medium (Thermo Fisher Scientific, Waltham, MA) and were preserved either in hot nematode fixative (70 % ethanol and 10 % glycerine) or in cold 3 % formalin. Worms were cleared in glycerol and examined as temporary mounts under the compound microscope for morphologic characters related to molting and sexual development. Drawings were made with a compound microscope Olympus BX51 (with differential interference contrast) and a drawing tube. Digital images were taken using differential interference contrast microscope Carl Zeiss Axio Imager 2 equipped with a digital camera Jenoptik ProgRes C7 and ProgRes CapturePro software. For examination of live worms, specimens were chilled briefly on ice and mounted in RPMI tissue culture media for light microscopy.

## Results

### Morphology of the L3

**Fig. 1:**
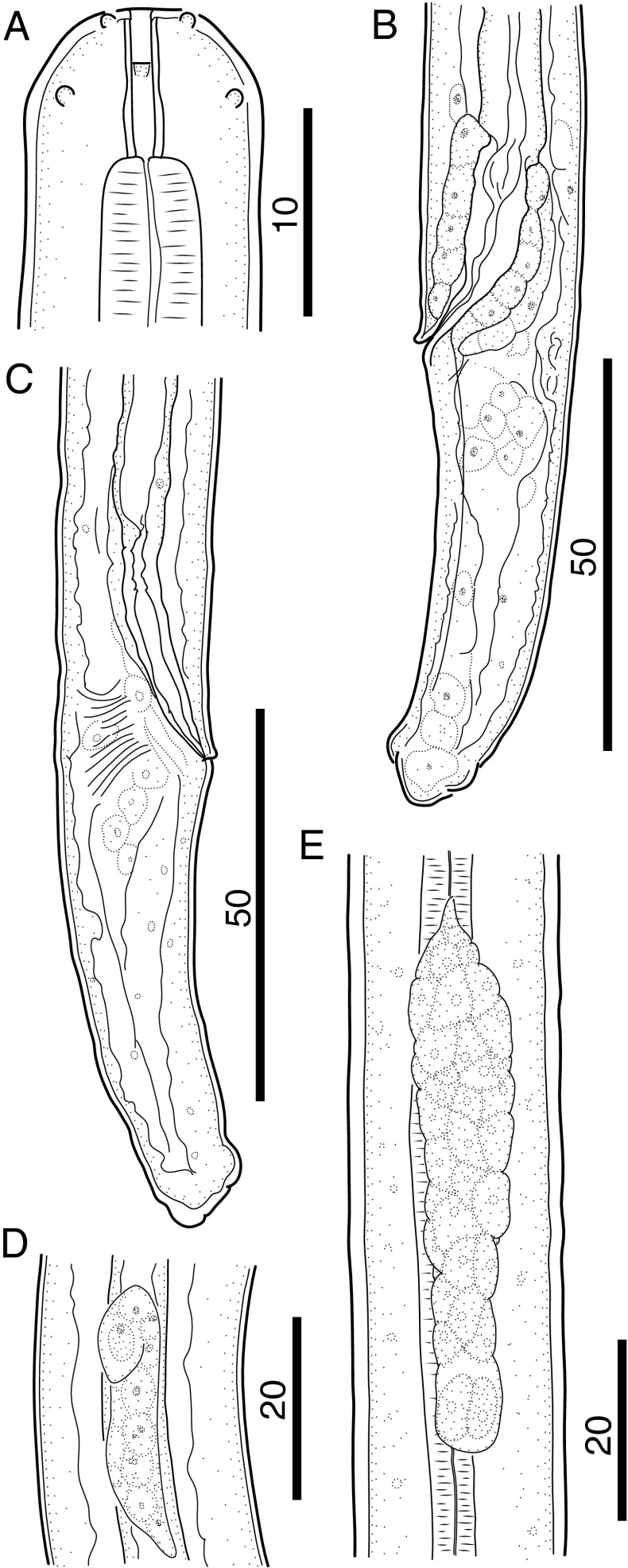
Morphology of third-stage larvae of *B. malayi*. **a** Cephalic extremity of 3 dpi larva, lateral view. **b** Posterior body end of 5 dpi male larva, lateral view. **c** Posterior body end of 5 dpi female larva, lateral view. **d** Anlage of reproductive system of 5 dpi male larva, lateral view. **e** Anlage of reproductive system of 7 dpi female larva, ventral view. *Scale bars* in micrometers

**Fig. 2:**
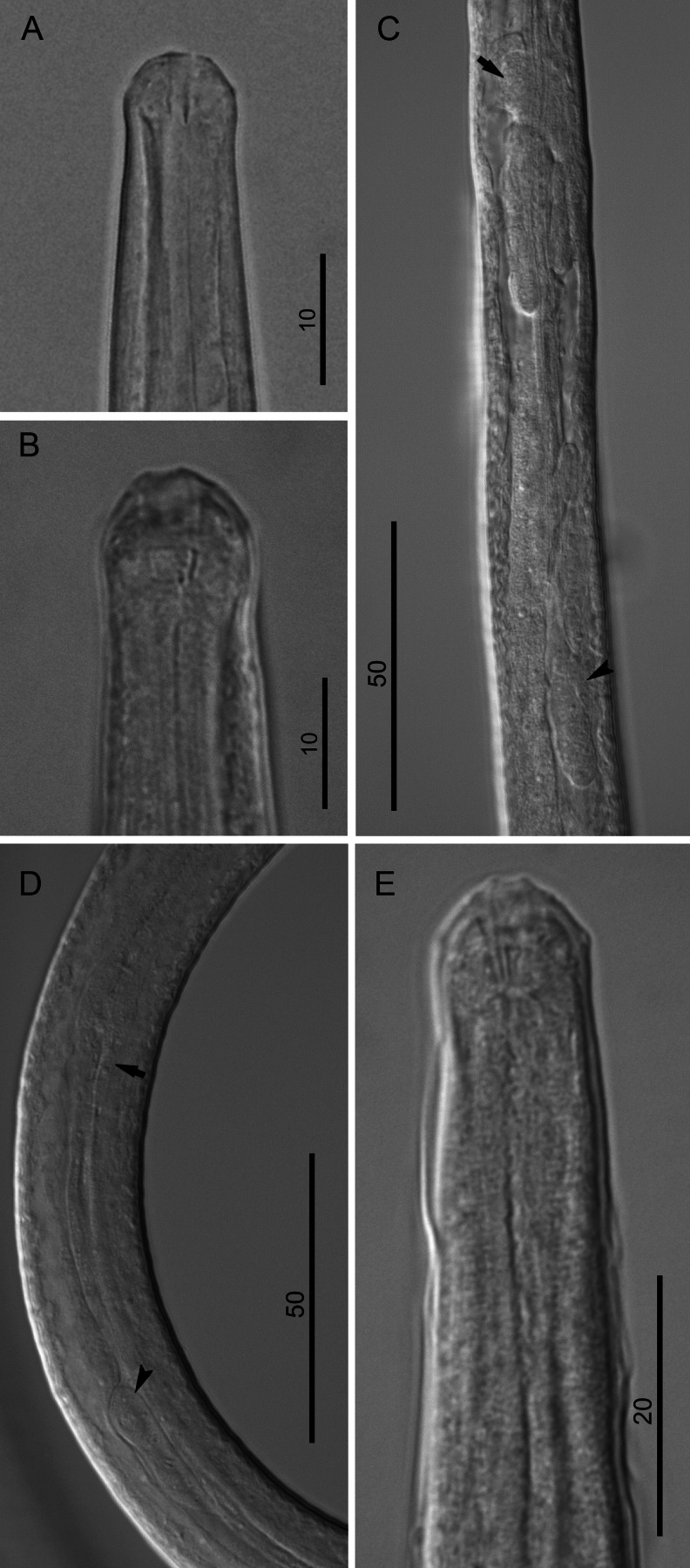
Morphology of third-stage larvae of *B. malayi*. **a** Cephalic extremity of 3 dpi larva, lateral view. **b** Cephalic extremity of 5 dpi larva, lateral view; note hyaline tissue around buccal cavity. **c** Anlage of reproductive system of 5 dpi female larva, right lateral view; note nerve ring (*arrow*) and genital anlage (*arrowhead*). **d** Anlage of reproductive system of 5 dpi male larva, lateral view; note esophago-intestinal junction (*arrow*) and genital anlage (*arrowhead*). **e** Anterior body end 7 dpi male larva, lateral view; note detached third-stage cuticle. *Scale bars* in micrometers

The characters most easily observed in L3 were the tapered head bearing eight submedian papillae arranged in two circles and a pair of lateral amphids, the long cylindrical buccal capsule (Figs. 1a and 2a) and the cuticular lappets on the tail extremity (Fig. 1b, c). In females, the genital primordium was located at the mid-esophagus, where it attaches to the body wall and begins to grow posteriorly (Figs. 1e and 2c). The male genital primordium was found just posterior to the esophago-intestinal junction (Fig. 2d), and its anterior growth turned back at 3 dpi, so that the anlage resembled a shepherds hook that grows posteriorly (Fig. 1d). After 4 dpi, thickening of the cuticle in the head region was observed in worms readying for the molt (Fig. 2b), and the excretory cell was visualized posterior to the nerve ring. In males, at 5 dpi the rectal wall was thickened in preparation for spicular pouch formation and male larvae were well distinct from those of female larvae (Fig. 1b). In females, the two ovarian precursor cells remained fused until the end of the third stage (Fig. 1e). Measurements of L3 are presented in Table 1.

**Table 1:** Measurements of *Brugia malayi* larvae recovered from the peritoneal cavity of jirds at various times after inoculation.  Measurements are in micrometers.

Days post infection (DPI)	Specimens studied	Body		Tail length	Anal body width	Distance head to testis reflection or vulva	Esophagus length	Tail length/body length	Esophagus length/body length	Distance head to testis reflection or vulva/body length
		Length	Width							
5	2♂, L3	1,681–1,756	25–27	58–70	19	618–721	545–616	0.034–0.040	0.324–0.351	0.367–0.410
5	3♀, L3	1,775–2,013 (1,898)	24–26 (25)	56–61 (59)	18–20 (19)	218–242 (232)	495–619 (576)	0.030–0.031 (0.031)	0.279–0.325 (0.303)	0.120–0.123 (0.122)
5	2♂, L3	1,713–1,744	27–28	61–62	19	687–798	585–644	0.035–0.036	0.341–0.369	0.401–0.458
5	3♀, L3	1,831–2,025 (1,915)	27–28	55–59 (57)	16–20 (18)	218–250 (232)	582–659 (611)	0.027–0.032 (0.030)	0.308–0.325 (0.319)	1.116–0.124 (0.121)
8	5♀, L3	1,832–2,156 (1,962)	27–28	58–66 (61)	18–20 (19)	240–260 (249)	548–671 (597)	0.029–0.033 (0.031)	0.290–0.311 (0.304)	0.118–0.132 (0.127)
9	5♂, L4	2,594–3,019 (2,829)	35–38 (36)	68–78 (73)	25–26	702–938 (791)	575–700 (649)	0.023–0.028 (0.026)	0.198–0.252 (0.230)	0.243–0.321 (0.280)
9	5♀, L4	2,819–3,363 (3,065)	36–38 (37)	75–81 (79)	23–26 (25)	316–342 (330)	495–670 (602)	0.023–0.028 (0.026)	0.161–0.227 (0.197)	0.094–0.121 (0.108)
21	5♂, L4	5,738–7,172 (6,485)	50–57 (53)	101–119 (113)	34–36 (35)	800–928 (873)	744–961 (814)	0.017–0.018	0.104–0.148 (0.126)	0.122–0.154 (0.135)
21	5♀, L4	6,391–9,844 (8,243)	50–60 (55)	97–116 (105)	30–37 (34)	530–911 (742)	725–850 (795)	0.010–0.018 (0.013)	0.079–0.124 (0.098)	0.065–0.143 (0.093)
29	2♂, L4	7,438–7,922	61–63	111–113	39	795–879	645–732	0.014–0.015	0.087–0.092	0.107–0.111
30	5♂, adults	8,141–10,672 (9,247)	59–65 (63)	126–139 (132)	40–47 (43)	749–1,025 (911)	682–850 (751)	0.012–0.016 (0.014)	0.064–0.096 (0.082)	0.083–0.120 (0.099)
34	5♀, L4	9,141–12,828 (11,441)	67–76 (71)	106–124(115)	35–37 (36)	590–710 (620)	699–910 (772)	0.009–0.012 (0.010)	0.059–0.077 (0.068)	0.047–0.066 (0.055)
35	3♀, adults	12,609–13,688 (13,177)	72–78 (76)	110–134 (121)	36–37	440–950 (657)	713–1,060 (861)	0.009–0.010	0.054–0.077 (0.065)	0.033–0.069 (0.050)
65	5♂, adults	16,688–19,422 (18,000)	72–84 (77)	156–183 (164)	42–50 (46)	935–1,325 (1,097)	820–969 (895)	0.008–0.011 (0.009)	0.048–0.052 (0.050)	0.056–0.074 (0.061)
65	5♀, adults	31,797–39,875 (34,694)	113–168 (139)	163–179 (172)	42–49 (45)	556–725 (667)	985–1,030 (1,010)	0.004–0.006 (0.005)	0.026–0.032 (0.029)	0.017–0.023 (0.019)

Metrical data are given as the range, with the mean value in parentheses

### Morphology of the L4

**Fig. 3:**
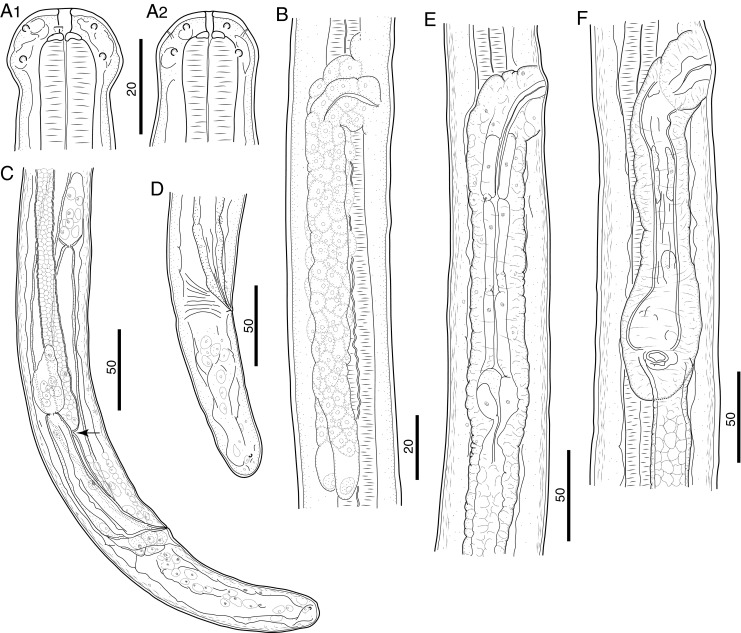
Morphology of L4 of *B. malayi*. **a** Cephalic extremity of 21 dpi larva, lateral and dorsoventral view, respectively. **b** Reproductive system of 9 dpi female larva, lateral view. **c** Posterior body end of 21 dpi male larva, lateral view; note developing vas deferens attached to rectum (*arrow*). **d** Posterior body end of 21 dpi female larva, lateral view. **e** Stage of development of vagina, 21 dpi larva, lateral view. **f** Stage of development of vagina, 31 dpi larva, lateral view. *Scale bars* in micrometers

**Fig. 4:**
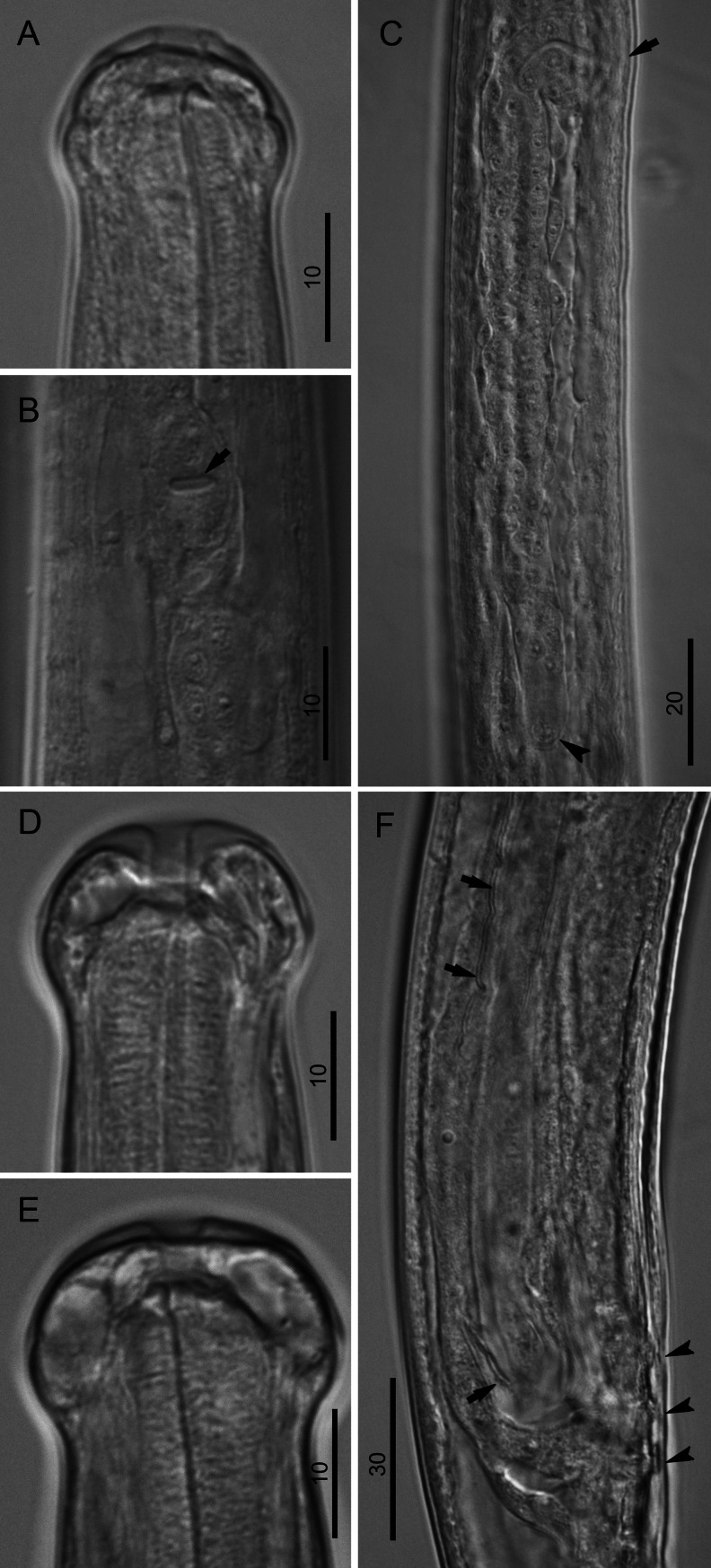
Morphology of L4 of *B. malayi*. **a** Cephalic extremity of 9 dpi, male larva, lateral view. **b** Region of vulva of 9 dpi larva, ventral view; note future canal of vagina (*arrow*). **c** Developing female reproductive system of 9 dpi larva, ventral view; note region of vulva (*arrow*) and precursor cell of ovary (*arrowhead*). **d** Cephalic extremity of 21 dpi, male larva, lateral view; note hyaline tissue around buccal cavity. **e** Cephalic extremity of 21 dpi, female larva, lateral view. **f** Posterior body end of 27 dpi male larva, lateral view; note development of caudal papillae (*arrowheads*) and right spicule (*arrows*). *Scale bars* in micrometers

Distinct characters of fourth-stage larvae (L4) were the globular head (bearing eight submedian papillae arranged in two circles and a pair of lateral amphids) and the buccal capsule that was characterized by a cylindrical anterior segment and thick posterior ring (Figs. 3a and 4a, d, e).

In male L4s as early as 10 dpi, the spicular pouches were not yet formed, but the rectal wall was thickened. Connection of the male genital system with the rectum to form the cloaca was not observed in our sampling, but in one 10 dpi larva that was 2,468 μm in length, a genital anlage of 1,289 μm was observed growing in posterior direction to 300 μm anterior to cloaca. This indicates that connection likely occurred soon after 10 dpi as observed by others (Schacher 1962). At 21 dpi, the vas deferens was attached to the rectum (Fig. 3c). At 27 dpi spicules, caudal papillae, and area rugosa were forming (Fig. 4f). In 29 dpi male larvae, the left and right spicules were not well developed, were weakly sclerotized, and measured 306–333 μm (*n* = 2) and 106–110 μm (*n* = 2) in length, respectively.

The visible but closed transverse vulva and the developing ovejector were readily apparent in an early stage of female L4 (Fig. 4b, c). In females, the genital anlage split into two tubes with visible terminal cap cells at 10 dpi (Figs. 3b and 4c). The terminal parts (vagina and ovejector) of the female reproductive system remained rectilinear till 30 dpi (Fig. 3b, e). At 30 and 31 dpi, all females were still in the L4 (*n* = 3 and 4, respectively), and at 31 dpi, three of four females were characterized by a distinct curve in the junction of vagina and ovejector (Fig. 3e).

Live 27 dpi L4 were examined using standard brightfield microscopy at ×200 and ×400 to identify visible characters in live worms. The prominent feature used to verify life cycle stage was the morphology of the buccal capsule. Because males and females do not differ greatly in length at this stage, characters used for sex determination were the presence/absence of a vulva and ovejector, of the single testis that is clearly fused with the cloaca in males, of the visible ends of the paired ovaries in the posterior part of females, and of the sclerotized gubernaculum in males. Spicules were not yet visible at this point in live worms and neither they nor the presence of a spirally curled tail could be used as an easily identifiable character for verifying sex. The larvae were sorted by sex and implanted IP in recipient jirds, the accuracy of sorting was verified when the worms reached adulthood (data not shown). In this case, it appeared that manipulation of L4s to determine stage and sex did not greatly affect their downstream survival after reimplantation into the peritoneal cavity of uninfected jirds. Measurements of L4 are presented in Table 1.

### Morphology of the adult

**Fig. 5:**
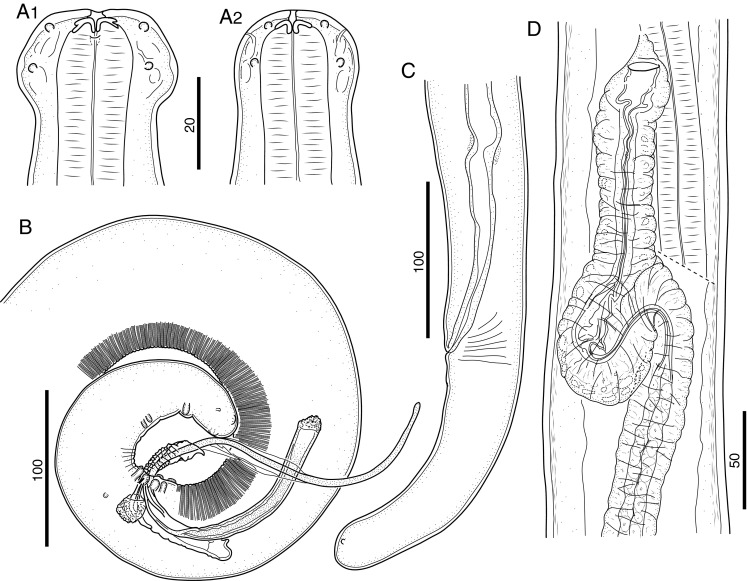
Morphology of adults of *B. malayi*. **a** Anterior body end of 65 dpi female, lateral and dorsoventral view, respectively. **b** Posterior body end of 176 dpi male, lateral view. **c** Posterior body end of 65 dpi female, lateral view. **d** Vagina of 65 dpi female, ventral view. *Scale bars* in micrometers

**Fig. 6:**
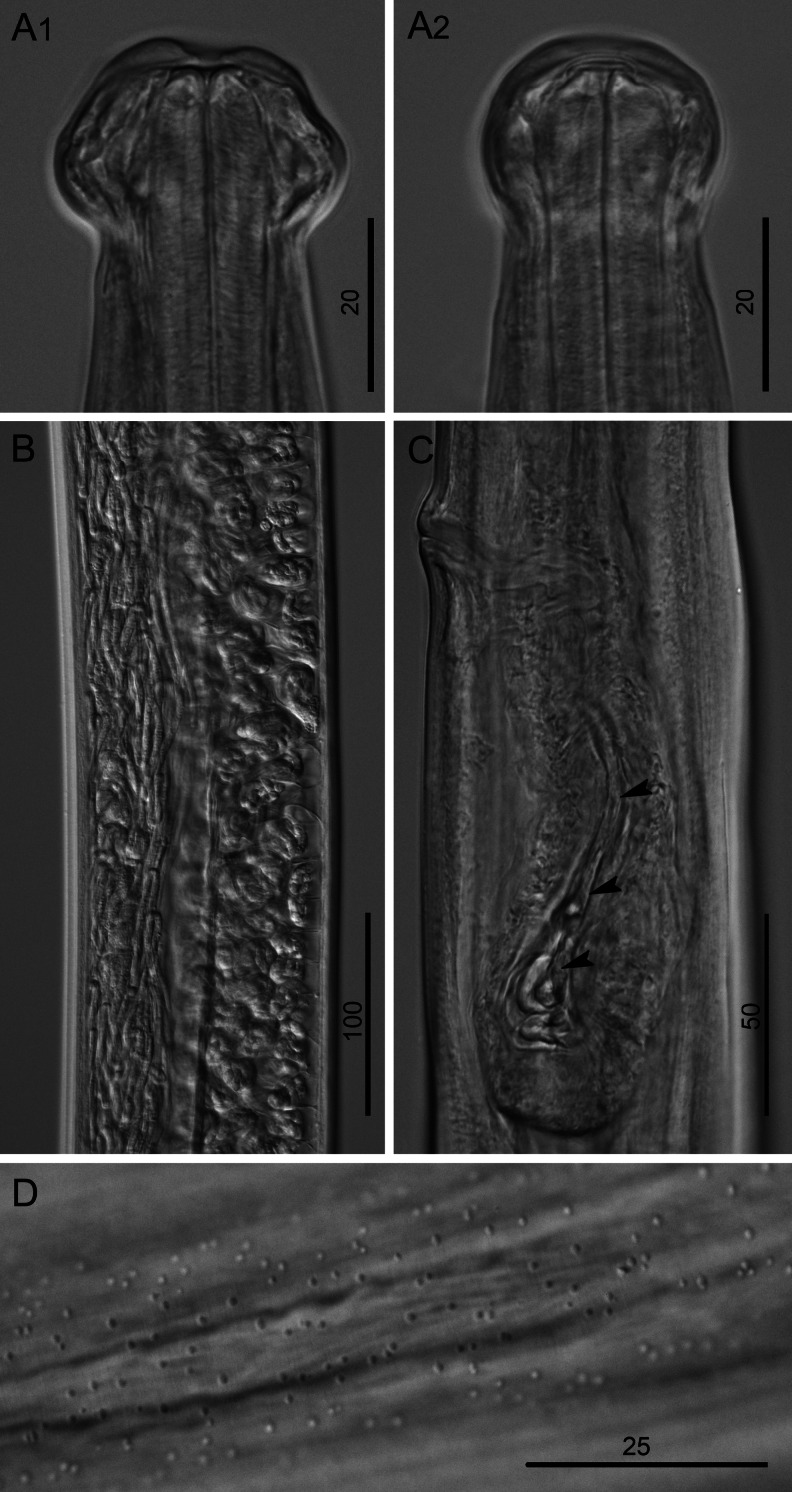
Morphology of 65 dpi female of *B. malayi*. **a** Cephalic extremity, lateral and dorsoventral view, respectively. **b** Region of uteri containing microfilariae and eggs. **c** Vagina with microfilaria (*arrowheads*), lateral view. **d** Dotted ornamentation on cuticle surface at posterior body end. *Scale bars* in micrometers

The adult worms were characterized by head morphology, bearing a distinct trapezoid shape in lateral view and rounded in dorsoventral view (Figs. 5a and 6a). The eight head submedian papillae arranged in two circles and the pair of lateral amphids were well distinct. The buccal cavity was formed by a shallow anterior portion and well-developed posterior ring. Males possessed prominent caudal papillae, area rugosa and sclerotized spicules (Fig. 5b). The left and right spicules in 30 dpi males measured 350–370 μm (mean, 361 μm; *n* = 5) and 111–121 μm (mean, 145 μm; *n* = 5) in length, respectively. They exhibited the same morphology and size as those of 65 dpi males (327–392 μm (mean, 367 μm; *n* = 5) and 106–121 (mean, 114 μm; *n* = 5)).

The cuticle surface at the posterior body extremity of the females differed from that of the males by the presence of irregular dotted ornamentation (Fig. 6d). The vulva was distinctly open and slightly protuberant in lateral view and was followed by the vagina and ovejector that had developed from a rectilinear structure to a coiled structure in adult females (Figs. 5d and 6c). The paired uteri met the ovaries in the posterior 1/3 of adult female worms. The vulva opened with the final molt, yet on 51 dpi, there were no sperm present in the oviducts, the uterine cells were swollen with prominent nuclei and nucleoli, and ovulae present in the posterior part of the uterus next to the oviduct were irregular-shaped and dead, demonstrating that mating had not yet occurred and egg production was not dependent on the presence of sperm. Measurements of adult worms are presented in Table 1.

### Temporal observations on development

**Fig. 7:**
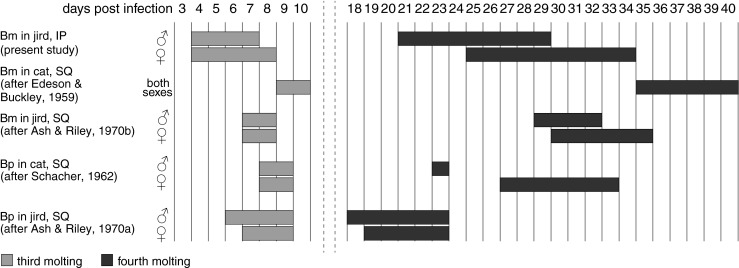
*Brugia* spp. development varies by host species and route of infection. *Bm B. malayi*, *Bp B. pahangi*, *IP* intraperitoneal, *SQ* subcutaneous. IP development of *B. pahangi* has not been reported

Modifications signifying the beginning of the third molt were evident on 4 dpi in male and female worms and were characterized by a thickening of the cuticle at the cephalic region, and the tail and peribuccal region became less defined and hyaline in appearance. Males molted slightly faster than females, at 7 dpi only males had loose cuticles (females *n* = 3 and males *n* = 2) and at 8 dpi four of six females had molted and the remaining two had loose cuticles while the only male examined had molted to the L4 (Fig. 2e). All worms examined were becoming L4 by day 9 (females *n* = 16 and males *n* = 11) (Table 1; Fig. 7). In males, the fourth molt started at 21 dpi (or possibly before) and ended on day 30, a span of at least 9 days. At 29 dpi, two molting male L4s were observed. At 30 dpi, six adult males and one molting male L4 were observed and at 31 dpi all males were young adults (*n* = 25). The fourth molt in females was first observed on 25 dpi. At 34 dpi, molting females were observed (*n* = 9), and by 35 dpi, all females had molted to young adults (*n* = 3). With respect to reproduction, young adult males at day 50 had spermatogonia forming in the testes and females at this time point were producing ova but no sperm were observed in the uteri indicating that mating had not yet occurred. At day 60 however, sperm was observed at the junction of the oviduct and the uterus in most females and dividing eggs were present. In a study of 24 adult females at 65 dpi only two contained eggs that were still at an early stage of development; the rest had MF in the uteri (Fig. 6b). Ten of these had MF in the ovejector (Fig. 6c). How quickly females are inseminated after sexual maturation *in vivo* is unknown, but in our experiments a 65-dpi virgin female that was placed in a culture dish for 2 h with multiple sexually mature males had sperm accumulated in the oviduct. This observation is consistent with findings that spermatozoa of *B. pahangi* could be found in adult females within 1 h of exposure to adult males *in vivo* (Burghardt and Foor 1975; Foor 1974). At 71 dpi, all females examined were fertilized and MF were present in the anterior uterus and vagina.

## Discussion

There are numerous laboratory animal models for filarial diseases, some that use permissive hosts for long-term infection and some with un permissive or manipulated hosts for short-term infection (Morris et al. 2013); the use of an appropriate model is dictated by the nature of the experiment (i.e. vaccinology, immunology, molecular biology). The Mongolian jird is a naturally permissive host for *Brugia* that when infected SQ develops some level of lymphatic pathology similar to what is seen with LF but generally does not develop clinical lymphadema. Conversely, IP - infected jirds lack significant pathology because the worms are confined mainly to the peritoneal cavity however this is the model of choice for large-scale worm production, , for example from 2010 to 2011 the NIH/NIAID Filariasis Research Reagent Resource Center (www.filariasiscenter.org) supplied over 9,000 IP-derived adult *B. malayi* and over 10,000 IP-derived *B. malayi* L4s to researchers in the USA and abroad; however, there were almost no requests for SQ infected animals in the same time period (Andrew Moorhead, personal communication).

Because the timeline of IP *Brugia* development in the jird was heretofore undescribed, it was historically necessary to presuppose it based on data from SQ *B. malayi* infections in cats and jirds (Ash and Riley 1970a; Edeson and Buckley 1959) or SQ *B. pahangi* infection in cats and jirds (Ash and Riley 1970b; Schacher 1962). The current study was designed to establish the timeline for *B. malayi* and to identify physical characters that are easily observed by compound light microscopy of live and fixed pre-adult worms to verify life cycle stage and sex. Detailed morphologic characterization of the mammalian stages of *Brugia* development can be found in previous reports (Buckley and Edeson 1956; Buckley 1960; Schacher 1962).

Unsurprisingly the majority of our observations relate to reproductive system development and molting. Molting is a complex and multi-stage process driven by enzymes that direct the degradation of the old cuticle (apolysis), synthesis of the underlying new cuticle, and separation of the old cuticle from the new (ecdysis or exuviation, often referred to as the molt) in an ordered series of events, while maintaining protection from the outside environment (Lee 2002). In between molts, the cuticle, a nonliving proteinaceous structure secreted from underlying hypodermal cells, is apparently continually remodeled to allow for growth of the worm, as evidenced in *Brugia* spp. by the dramatic increases in length observed in second stage larvae in the mosquito vector and early adults in the mammalian host... Molting of the external cuticle is also accompanied by casting of the amphid, phasmid, buccal cavity, esophageal, rectal, and *vagina vera* cuticular linings and is likely mediated by neurosecretory hormones such as ecdysteroids (Lee 2002). For the purposes of our study, the first morphological signs signifying the beginning of the molt were observed in the cephalic region, where the apical cuticle became slightly thicker and the peribuccal region became less defined. Thickening of the cuticle over the entire body and separation of the old and new buccal capsules followed these events and dramatic loosening of the old cuticle from the body was observed on the last day preceding exuviation.

Using these criteria, we have shown that the timing of larval development of *B. malayi* differs between the IP and SQ jird models. The fourth molt for males and females occurred earlier and was spread over more days than was previously reported for SQ *B. malayi* in jirds (Ash and Riley 1970a), and both molts occurred considerably earlier than in SQ infected cats (Edeson and Buckley 1959). The third molt of IP *B. malayi* in jirds started and ended before that of its sister species *B. pahangi* in SQ cats and jirds, but the fourth molt of IP *B. malayi* in jirds occurred considerably later than that of *B. pahangi* in SQ jirds. There are no reports on the timing of IP *B. pahangi* in jirds that could be used for comparison. It is unlikely that the size of the inoculum affected the developmental timeline in our experiment because it was previously shown that 5-fold differences in inoculum (20–100 L3/animal) did not affect the timing of prepatency in male jirds (el-Bihari and Ewert 1973). We propose that a similar developmental study of IP *B. pahangi* in jirds could also be of great utility in characterizing filariasis models.
